# Outcomes and Prognostic Markers in Extracorporeal Cardiopulmonary Resuscitation: 10-Year Experience from a Rural Tertiary Care Center

**DOI:** 10.3390/diagnostics15101275

**Published:** 2025-05-17

**Authors:** Kamran Namjouyan, Aastha Mittal, Evan Gajkowski, Amanda Young, Sudheer Penupolu, Brendan Carry

**Affiliations:** 1 Medicine Institute, Geisinger Medical Center, Danville, PA 17821, USA; 2 Critical Care Institute, Geisinger Medical Center, Danville, PA 17821, USA; 3 Heart Institute, Geisinger Medical Center, Danville, PA 17821, USA; 4 Biostatistics Institute, Geisinger Medical Center, Danville, PA 17821, USA

**Keywords:** refractory cardiac arrest, extracorporeal cardiopulmonary resuscitation, prognostic markers

## Abstract

**Background:** Extracorporeal cardiopulmonary resuscitation (eCPR) is a method for initiation of cardiopulmonary bypass during resuscitation of a patient with refractory cardiac arrest to support end-organ perfusion. This retrospective study evaluates which prognostic markers are seen in patients with poor outcomes who underwent eCPR in our rural tertiary care center. **Study Design/Methods:** All patients who underwent eCPR at our center from May 2013 to January 2023 were analyzed in a retrospective manner. We then compared outcomes in patients who survived to discharge (survivors) versus those who did not survive to discharge (non-survivors). Demographic factors, body mass index, peak serum lactate in 24 h, initial rhythm, lowest mean arterial pressure within the first six hours, a requirement of renal replacement therapy, and the number of blood transfusions required during the hospitalization were analyzed. **Results:** 37 patients (24 males and 13 females) with a median age of 58 years (IQR: 48–65) were included. The overall mortality rate was 75.7%, and all survivors had good neurological outcomes, which were defined as Cerebral Performance Category (CPC) scores of 1 or 2. The most significant factors seen in non-survivors were obesity as measured by BMI more than 30 (odds ratio = 7.33; 95% CI 1.40–38.33; *p* = 0.02), and lowest MAP <65 within the first 6 h despite being on extracorporeal membrane oxygenation (0% vs. 74.1%; *p* = <0.01). **Conclusions:** This retrospective study demonstrates that initial presentations of patients who underwent eCPR with obesity and MAPS < 65 within the first 6 h despite ECMO support were seen in patients with higher mortality.

## 1. Introduction

Cardiac arrest is one of the leading causes of death worldwide, with a prevalence of >500,000 in the United States and a survival rate of <15% [1]. Despite decades of research and many new technologies, high-quality Cardiopulmonary Resuscitation (CPR) remains the single best method of resuscitation [2]. However, even with the highest-quality CPR, only a third of normal blood flow to the heart and brain can be delivered [1]. This further emphasizes the need to train rescuers to deliver the best quality CPR possible for a better chance of achieving the return of spontaneous circulation (ROSC). However, despite all interventions, mortality rates are between 50 and 60%, and many do not achieve full neurological recovery upon discharge [3].

Refractory cardiac arrest is characterized by extended CPR efforts, or more than three attempts at defibrillation without achieving ROSC. Utilization of conventional pharmacological interventions such as antiarrhythmics or pressors has failed to demonstrate any significant impact on the survival rates of patients with refractory cardiac arrest. Despite advancements in the approach to cardiac arrest, refractory cardiac arrest remains one of the most challenging encounters for physicians, necessitating further need to explore treatment strategies to improve outcomes for affected patients. Therefore, many new technologies such as mechanical CPR devices, targeted temperature management, and Extracorporeal Cardiopulmonary Resuscitation (eCPR) have been developed [4].

eCPR is an effective but invasive procedure that utilizes cardiopulmonary bypass during the resuscitation of patients experiencing refractory cardiac arrest. eCPR aims to sustain end-organ perfusion while underlying possible reversible conditions have been assessed and treated. Meta-analyses have shown a wide spectrum of survival rates, ranging from 8% to 50%, alongside varying ranges of neurological outcomes among patients who have undergone eCPR. Comparatively, when conventional therapy is weighed against eCPR, it has shown a notable 13% absolute increase in 30-day survival rates [3].

According to data from the Extracorporeal Life Support Organization (ELSO) registry, a tenfold increase in the utilization of eCPR over the past decades has been observed, underlining eCPR’s increasing recognition and adoption in many health care centers. Notably, the ARREST trial demonstrated a significant improvement in survival to discharge when early eCPR was employed for patients experiencing refractory ventricular fibrillation and out-of-hospital arrest [5]. Comparatively, recent randomized clinical trials showed that early utilization of eCPR as compared to conventional therapy in refractory out-of-hospital cardiac arrest was associated with similar survival rates but favorable neurological outcomes [6,7]. While eCPR remains an effective strategy for a subset of patients experiencing refractory cardiac arrest, it remains unclear whether the patient population benefits the greatest from this invasive procedure, warranting further investigation.

Given the discrepancy and uncertainty in the data about the benefits of using this highly complex method of resuscitation, a multidisciplinary approach is needed to select patients for such interventions. Our aim is to conduct a retrospective analysis of patients who have undergone this procedure in our rural tertiary care center, with a goal of recognizing prognostic factors linked to unfavorable outcomes in patients with refractory cardiac arrest. eCPR is a modality that is significantly expensive and has been linked to many complications, including thoracic or abdominal hemorrhage, along with many others. This study hopes to provide clinicians with further insights into the uncertainties of eCPR and assist them in making informed decisions regarding the utilization and stratification of patients for this invasive supportive modality.

## 2. Materials and Methods

### 2.1. Study Setting

This study pursued a retrospective analysis of all patients suffering from refractory cardiac arrest who underwent eCPR at our rural tertiary care center located in Central Pennsylvania from May 2013 to January 2023. Within our facility, when a provider identifies a potential case of refractory cardiac arrest, whether in-hospital or out-of-hospital, they will alert the ECMO provider on call to evaluate the patient’s suitability for eCPR. The ECMO provider evaluates the case with a comprehensive review of the patient’s profile, encompassing factors such as age, co-morbidities, and pertinent laboratory findings.

### 2.2. Patient Selection

**Figure 1:**
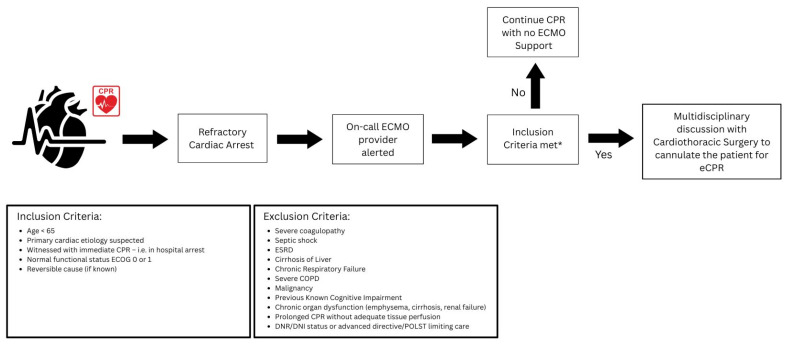
The eCPR protocol at our rural tertiary care center utilizing ECMO to provide advanced life support during cardiac arrest.

The eligibility criteria within our facility for eCPR include being under the age of 65, primary cardiac etiology suspected, witnessed cardiac arrest with immediate CPR (i.e., in hospital arrest), normal functional status (as defined by everyday Cognition (ECog) score 0 or 1), and a reversible cause (if known). Patients with the following conditions are not eCPR candidates: severe coagulopathy, septic shock, end-stage renal disease, cirrhosis of the liver, chronic respiratory failure, severe COPD, malignancy, previous known cognitive impairment, and advanced directives stating DNR/DNI status. After a review of the patient by the ECMO provider, if the patient does not have any significant contraindications to ECMO support, the case is further discussed with the cardiothoracic surgery team, who proceed to cannulate the patient. The ECMO team, including a critical care physician, then oversees patients’ transfer to the cardiac intensive care unit post-ROSC (Figure 1).

### 2.3. Clinical Outcomes

After careful chart review, a total of 37 individuals met the inclusion criteria, having undergone resuscitation with eCPR in conjunction with conventional CPR within our tertiary care center. We then analyzed and compared outcomes in patients who survived to discharge (survivors) versus those who did not survive discharge (non-survivors). Furthermore, we investigated the neurological outcomes of survivors based on the Cerebral Performance Category (CPC) scores. We defined good neurological outcomes (GNOs) as CPC scales of 1 and 2. Poor neurological outcomes (PNOs) were defined as CPC scales of 3, 4, and 5.

A literature review of all publications from 2010 to 2023 was conducted using the MEDLINE database on the OVID platform. Keywords that were used in the search were “eCPR outcomes”, “eCPR prognostic makers”, etc. Several reported prognostic factors based on smaller studies including demographic factors (age, sex, and gender), body mass index (BMI), initial rhythm, duration of cardiac arrest, peak serum lactate in 24 h, initial rhythm, lowest mean arterial pressure (MAP) within first six hours, a requirement of renal replacement therapy, and the number of blood transfusions required during the hospitalization were analyzed.

### 2.4. Statistical Analysis

Variables were summarized using frequencies and percentages or median and interquartile range (IQR), depending on the data. Comparisons were between mortality (no—survived to discharge, yes—did not survive to discharge) using the Chi-square test statistic for categorical variables and Kruskal–Wallis test for continuous outcomes. Logistic regression was used to assess the odds of mortality, both unadjusted and then adjusted for age. Odds ratios and associated 95% confidence intervals (CIs) and *p*-values were reported. *p*-values less than 0.05 were considered significant. All analyses were performed using SAS v9.5 (SAS Institute, Inc., Cary, NC, USA). This protocol was approved by the Geisinger Institutional Review Board on 29 March 2023, under IRB number 2023-1066.

## 3. Results

This retrospective cohort study included 37 patients (24 (64.9%) males and 13 (35.1%) females), of whom the median age was 58 years (IQR: 48–65). The overall mortality rate was 75.7%. All the survivors (9 patients; 24.3%) had GNO, as detailed in Table 1 and Table 2.

The duration of cardiac arrest was defined as the start of CPR upon first medical contact. The median duration of cardiac arrest was 60 min (IQR; 15, 60) for non-survivors as compared to 10 min (IQR; 10, 20) for survivors (*p* = 0.01). The median duration on ECMO support was 1.9 days (IQR; 0.5, 7.8) for the non-survivors as compared to 4.8 days (IQR; 1.8, 7.9) for the survivors, *p* = 0.35, as outlined in Table 3.

Obesity as measured by BMI greater than or equal to 30 was statistically significant between the two groups with an odds ratio of 7.33; 95% CI: 1.40–38.33; *p* = 0.02. Given that age was significant between the two groups, we adjusted for age, and obesity continued to show association with survivability, with an odds ratio of 12.13, 95% CI: 1.57–93.46; *p* = 0.02. Careful analysis of blood pressure readings and MAPs while admitted was performed and showed that MAPs < 65 within the first 6 h despite being on ECMO was noted to be a significant marker associated with mortality, 0% vs. 74.1%; *p* = <0.01. However, this was not statistically significant when adjusted for age, *p* = 0.93.

There was no significant association between mortality and non-shockable rhythm (odds ratio = 5.09; 95% CI: 0.89–29.26; *p* = 0.07), peak serum lactate in 24 h >10 mmol/L (odds ratio = 4.90; 95% CI: 0.84–28.72; *p* = 0.08), use of continuous renal replacement therapy (CRRT) (odds ratio = 1.15; 95% CI: 0.25–5.34; *p* = 0.80) and individuals requiring more than ten blood transfusions (odds ratio = 1.11; 95% CI: 0.23–5.43; *p* = 0.90), as detailed in Table 4.

## 4. Discussion

eCPR remains a critical emergency measure to potentially improve survival among patients suffering from refractory cardiac arrest. The decision to initiate eCPR is always in an acute setting, and often clinicians operate within minutes, having minimal data available in determining which subset of patients would have the greatest benefits from this aggressive approach. Moreover, this decision is further complicated due to no specific guidelines available to delineate which patient population benefits the most from the use of this aggressive intervention. According to the American Heart Association (AHA) guidelines, eCPR may be considered for select cardiac arrest patients with potentially reversible causes during a limited period of mechanical circulatory support [8]. The European Association for Cardiothoracic Surgery, Society of Thoracic Surgeons, American Association for Thoracic Surgery, and Extracorporeal Life Support Organization also emphasize the importance of patient selection criteria, including observed cardiac arrest, presumed cardiac etiology, short no-flow and low-flow times, and the presence of reversible causes [9].

Recent clinical trials have provided some insights into the benefits and efficacy of early eCPR initiation. The ARREST trial underscores the superiority of early eCPR initiation as compared to conventional therapy. Additionally, the INCEPTION trial, although it found similar results in mortality between the two groups, it did find favorable neurological outcomes in the eCPR group as compared to the conventional therapy, but the difference did not reach statistical significance, likely due to the small sample size [5,6]. Despite many facilities incorporating a multidisciplinary approach to select patients for such interventions, the decision to initiate eCPR continues to be highly complex.

This study analyzed several factors and their correlation with mortality prior to discharge. The primary endpoint was mortality, which was observed at a rate of 75.7%. Notably, this slightly exceeds the national mortality rate of eCPR per ELSO registry, which stands at 70% [5]. It is notable to report that all the survivors had GNO. There was a statistical difference between the two groups’ ages, with a median age of 61 among non-survivors compared to 46 among survivors. Furthermore, the median duration of cardiac arrest was 60 min in the non-survivor group as compared to 10 min in the survivor group, a statistically significant difference with a *p*-value of 0.01. Moreover, the mean duration on ECMO was shorter for patients who did not survive to discharge as compared to the survivor group, 1.9 vs. 4.8 days, *p*-value of 0.35. These findings could signify a sicker and older population in the non-survivor group. Furthermore, this study did not account for the patients who might have been transitioned to comfort care based on overall prognosis and goals of care in those specific scenarios.

Several studies have investigated age as a marker of survivability, specifically in those aged 65 and older. It was found that older individuals have significantly lower survival rates and poorer neurological outcomes compared to younger patients. For instance, patients aged 65–74 and those older than 75 have decreased odds of survival compared to those aged 18–49 [10,11]. The AHA further suggests that an age greater than 75 years is generally unfavorable for eCPR. Moreover, prior studies support our finding that the duration of cardiac arrest, particularly the low-flow duration (time from cardiac arrest to initiation of ECMO), is a critical determinant of eCPR outcomes [12,13]. These studies are in accordance with our findings for this patient population.

This study further delved into all prognostic markers reported in the literature and applied them to our center’s eCPR dataset. Analysis showed that obesity, as measured by BMI greater than or equal to 30, was statistically significant between the two groups, regardless of age. The authors hypothesize that obesity leads to worse outcomes due to procedural challenges with cannulation and higher rates of wound infection, although this was not further investigated due to the retrospective nature of the study. These factors can complicate treatment and recovery, contributing to poorer overall outcomes. This is particularly important given that the decision to initiate eCPR usually occurs with minimal data available, and this perhaps offers a valuable data point that is easily available by reviewing health records. Several studies have demonstrated that higher BMI is associated with worse outcomes in eCPR. For instance, Kosmopoulos et al. found that patients with a BMI > 30 kg/m^2^ had significantly lower survival to hospital discharge compared to those with a BMI ≤ 30 kg/m^2^ (29.3% vs. 48%, *p* < 0.001). This study also identified BMI as an independent predictor of mortality in a multivariable logistic regression analysis [14]. Similarly, Pai et al. reported a U-shaped relationship between BMI and mortality, with class III obesity (BMI ≥ 35 kg/m^2^) being significantly associated with higher mortality in the ECPR subgroup (hazard ratio 2.71, 95% CI 1.71–4.29, *p* < 0.001) [15]. Additionally, Beni et al. found that obesity was associated with greater odds of in-hospital mortality in pediatric patients undergoing ECPR (adjusted odds ratio 2.28, 95% CI 1.21–4.31) [16].

Furthermore, this study also found that MAPs < 65 within the first 6 h despite being on ECMO was noted to be a significant marker seen in non-survivors, although this was not statistically significant when adjusted for age. While the exact reason for this factor losing significance when adjusted for age remains unclear, the authors hypothesize that the relatively young age of both groups may have mitigated its impact on the outcome. Several studies have demonstrated the importance of maintaining an adequate MAP during ECMO to ensure favorable outcomes. Lee et al. found that patients with an average MAP below 60 mmHg had a high probability of poor neurological outcomes, while those with an average MAP around 75 mmHg had the least probability of poor outcomes [17]. Similarly, Ryu et al. identified an initial MAP less than 70 mmHg as a significant predictor of poor neurological outcomes in patients undergoing eCPR [18]. Notably, both markers (Low MAPs and BMI) are available early in the hospital course and can possibly help clinicians in determining prognostic assessment and treatment strategies, but further studies will need to confirm this finding, given our small sample size.

It has been reported in a systematic review analysis that non-shockable rhythm was noted to be associated with higher mortality rates in eCPR, but in our cohort, this was not found to be a significant factor [19]. However, it is worth noting that the sample size could influence this finding. Upon closer analysis, it was noted that out of the 18 patients with non-shockable rhythm, 16 did not survive, while only 2 survived to discharge, yielding an odds ratio of 5.09. This finding could perhaps become statistically significant with a larger sample size, given the trend seen in our analysis. Several studies have shown that an initial shockable rhythm, like ventricular fibrillation or pulseless ventricular tachycardia, is linked to better survival rates and neurological outcomes in ECPR patients. For example, Wang et al. found that an initial shockable rhythm significantly predicts both survival (odds ratio [OR] 2.29, 95% confidence interval [CI] 1.53–3.42) and favorable neurological outcomes (OR 2.33, 95% CI 1.20–4.52) in their meta-analysis [20]. Additionally, Daou et al. found that initial shockable rhythm was an independent predictor of favorable neurological outcomes in patients undergoing ECPR (OR 9.64, 95% CI 1.49–62.30) [21].

Serum lactate has emerged as a marker commonly utilized in such patients to further assist with the candidacy for eCPR. In our study, the lactate levels were carefully tracked during the hospitalization while on ECMO. Notably, analysis showed a difference of 2 patients as opposed to 14 who survived to discharge with lactates greater than 10 in 24 h. This observation further supports that serial lactate is perhaps a meaningful prognostic marker that can assist clinicians, along with other markers, to determine the prognosis. Multiple studies have highlighted the prognostic significance of serum lactate levels and lactate clearance in eCPR patients. Thevathasan et al. discovered that both pre-ECPR lactate levels and 24 h lactate clearance were strongly linked to one-year survival in a large, multicenter cohort study, with lower pre-ECPR lactate levels and higher 24 h lactate clearance correlating with better survival outcomes [22]. Similarly, Sugimoto et al. emphasized the importance of lactate clearance, demonstrating that higher modified 6 h lactate clearance was associated with improved 30-day survival and favorable neurological outcomes in patients with out-of-hospital cardiac arrest treated with ECPR [23].

While this study acknowledges the main limitation, which is the sample size of 37, it is crucial to emphasize the intricate nature of eCPR. This is a highly complex intervention demanding close collaboration among different specialties, a resource not readily accessible in many healthcare facilities. Furthermore, our literature review revealed a consistent trend that the majority of eCPR clinical trials and retrospective studies have been conducted on notably small sample sizes. Moreover, this study was not able to separate in-hospital and outside-hospital cardiac arrests. Additionally, time to cannulation was not documented in our record system, and this factor has been reported as a significant finding in previous studies; therefore, careful attention should be given to this value in future studies if data are available. For the next steps, we recommend pursuing a larger sample size, preferably in a multi-center setting, to validate and confirm the above-observed prognostic factors.

## 5. Conclusions

The survival rate of patients who undergo eCPR remains poor, and careful consideration should be given in selecting the patients for such intervention [24]. The probability of having good neurological outcomes should be taken into consideration while selecting the patients for eCPR. This study presents an analysis of all patients in our tertiary care center who underwent eCPR and offers a comprehensive review of available prognostic markers in the literature. This study demonstrated that initial presentations including obesity and MAPS < 65 within the first 6 h despite ECMO support (although not statistically significant when adjusted for age) were the most significant prognostic markers potentially seen in patients with higher mortality, and therefore careful study of the factors discussed above should be considered for such patients. Further studies are indicated to evaluate whether such prognostic markers continue to hold a strong association in a larger population.

## Figures and Tables

**Table 1:** Generalized study population data.

Total Patients	37
**Age, Median (IQR)**	58 (48, 65)
**Male**, n (%)	24 (64.9%)
**CPC Score**	
**Good Neurological Outcomes (CPC 1 &2)**	9 (24.3%)
**Poor Neurological Outcomes (CPC 3,4 &5)**	28 (75.7%)

**Table 2:** Age and gender comparison among survivors and non-survivors (^1^ Kruskal–Wallis *p*-value; ^2^ Chi-square *p*-value).

	Mortality		
	No	Yes	*p*-Value
	(N = 12)	(N = 25)	
**Age, Median (IQR)**	46 (35, 53.5)	61 (51, 72)	<0.01 ^1^
**Male**, n (%)	8 (66.7%)	16 (64.0%)	0.87 ^2^

**Table 3:** Duration of cardiac arrest and ECMO support (^1^ Kruskal–Wallis *p*-value; ^2^ Chi-square *p*-value).

	Poor Neurological Outcomes (N = 28)	Good Neurological Outcomes (N = 9)	Odds Ratio (95% Cl)	*p*-Value
**Duration of cardiac arrest less than 15 min, n (%)**	5/23 (21.7%)	4/7 (57.1%)	4.80 (0.80–28.90)	0.09 ^1^
**Duration of cardiac arrest in minutes, Median (IQR)**	60 (15, 60)	10 (10, 2)	N/A	0.01 ^2^
**Time on ECMO in days, Median (IQR)**	1.9 (0.5, 7.8)	4.8 (1.8, 7.9)	N/A	0.35 ^2^

**Table 4:** Prognostic markers among patients who survived to discharge versus non-survivors (^1^ Wald Chi-square *p*-value; ^2^ Kruskal–Wallis *p*-value).

		Mortality				Age Adjusted	
	Total	No	Yes	Odds Ratio (95% CI)	*p*-Value	Odds Ratio (95% CI)	*p*-Value
	(N = 37)	(N = 9)	(N = 28)				
**Non-shockable rhythm**, n (%)	18 (50.0%)	2 (22.2%)	16 (59.3%)	5.09 (0.89–29.26)	0.07 ^1^	3.79 (0.60–23.75)	0.07 ^1^
Missing	1	0	1				
**Obese**, n (%)	25 (67.6%)	3 (33.3%)	22 (78.6%)	7.33 (1.40–38.33)	0.02 ^1^	12.13 (1.57–93.46)	0.02 ^1^
**MAP in first 6 h, Median (IQR)**	62.5 (54.0, 70.0)	70 (65, 80)	60 (50, 65)		<0.02 ^2^		
Missing	1	0	1				
**MAP in first 6 h < 65**, n (%)	20 (55.6%)	0 (0.0%)	20 (74.1%)	N/A	<0.01 ^1^	N/A	0.93 ^1^
Missing	1	0	1				
**Peak lactate in 24 h, Median (IQR)**	9.2 (6.5, 14.0)	6.9 (3.6, 9.0)	11.2 (7.2, 14.7)		0.06 ^2^		
Missing	4	0	4				
**Peak lactate in 24 h > 10**, n (%)	16 (48.5%)	2 (22.2%)	14 (58.3%)	4.90 (0.84–28.72)	0.08 ^1^	6.97 (0.91–53.52)	0.06 ^1^
Missing	4	0	4				
**Need for RRT**, n (%)	16 (47.1%)	4 (44.4%)	12 (48.0%)	1.15 (0.25–5.34)	0.80 ^1^	1.19 (0.23–6.26)	0.84 ^1^
Missing	3	0	3				
**Blood transfusion, Median (IQR)**	6 (0, 16)	6 (5, 17)	6 (0, 16)		0.55 ^2^		
**Blood transfusion > 10**, n (%)	13 (35.1%)	3 (33.3%)	10 (35.7%)	1.11 (0.23–5.43)	0.90 ^1^	0.84 (0.15–4.81)	0.85 ^1^

## Data Availability

The original contributions presented in this study are included in the article. Further inquiries can be directed to the corresponding author.

## Abbreviations

AHA	American Heart Association
BMI	Body Mass Index
CPC	Cerebral Performance Categories
eCPR	Extracorporeal Cardiopulmonary Resuscitation
ECMO	Extracorporeal Membrane Oxygenation
GNO	Good Neurological Outcomes
IQR	Interquartile Range
MAP	Mean Arterial Pressure
PNO	Poor Neurological Outcomes
ROSC	Return of Spontaneous Circulation
